# A multidrug-resistant *Klebsiella pneumoniae* outbreak in a Peruvian hospital: Another threat from the COVID-19 pandemic

**DOI:** 10.1017/ice.2020.1401

**Published:** 2021-01-05

**Authors:** Kovy Arteaga-Livias, Karim Pinzas-Acosta, Lourdes Perez-Abad, Vicky Panduro-Correa, Ali A. Rabaan, Samuel Pecho-Silva, Bernardo Dámaso-Mata

**Affiliations:** 1 Hospital II-EsSalud, Huánuco, Perú; 2 Facultad de Medicina, Universidad Nacional Hermilio Valdizán, Huánuco, Perú; 3 Universidad Científica del Sur, Lima, Perú; 4 Hospital Nacional Dos de Mayo, Lima, Perú; 5 Molecular Diagnostic laboratory, Johns Hopkins Aramco Healthcare, Dhahran, Saudi Arabia

*To the Editor*—Since the report of the first cases of coronavirus disease 2019 (COVID-19), attempts have been undertaken to contain its expansion; however, to date there have been >60 million infections and ∼1.5 million people have died. This pandemic has challenged all knowledge about public health in countries and institutions around the world, and in addition to the morbidity and mortality of the disease, other conditions have appeared that threaten public health.^1^

One of these situations that has not received adequate attention is the increase in bacterial resistance and the emergence of multidrug-resistant (MDR) strains, probably associated with multiple factors such as the collapse of health systems, self-medication of the population, indiscriminate use of antibiotics in hospitals, a false sense of security, and incorrect use of personal protective equipment (PPE).^2^

We reported an outbreak of *Klebsiella pneumoniae* New Delhi metallo-β-lactamase (NDM) in a Peruvian hospital where no cases of strains with this resistance had been identified previously. All patients were admitted for a diagnosis of COVID-19 and were placed in isolation and management areas for treatment. Table 1 shows the clinical characteristics of the patients included in the study.

**Table 1.** Clinical Characteristics of Patients With Positive Culture for Multidrug-Resistant *Klebsiella pneumoniae*

Characteristic	Patient 1	Patient 2	Patient 3	Patient 4
Age, y	62	66	45	52
Sex	Male	Male	Male	Male
Medical history	High blood pressure, hypothyroidism	None	Obesity	None
Time since onset, d	7	14	8	12
Previous medication	Hydroxychloroquine Amoxicillin/ clavulanic acid	Ceftazidime Ciprofloxacin	Ceftriaxone	Ceftriaxone Azithromycin
PaO_2_/FiO_2_ on entry	191	150	178	200
Diagnosis on entry	Type 1 ARF Moderate COVID-19 ARDS	Type 1 ARF Severe COVID-19 ARDS	Type 1 ARF Moderate COVID-19 ARDS	Type 1 ARF Moderate COVID-19 ARDS
Hospital entry date	Aug 21, 2020	Aug 16, 2020	Aug 14, 2020	Aug 27, 2020
Days hospitalized prior to ICU admission	4	6	19	1
Date of ICU entry	Aug 25, 2020	Aug 22, 2020	Sept 3, 2020	Aug 28, 2020
Treatment received in ICU	Ceftazidime Ciprofloxacin Colistin Gentamicin	Imipenem Vancomycin Gentamicin Nitrofurantoin	Imipenem Vancomycin Ertapenem Clarithromycin	Piperacillin/ Tazobactam Gentamicin Colistin
Date of endotracheal secretion culture	Sept 13, 2020	Sept 2, 2020	Sept 13, 2020	Sept 7, 2020
Days in ICU	29	27	18	11
Superimposed infection	*Acinetobacter baumannii*	None	*Serratia marcescens*	None
Resistance genes	NDM CTX-M	NDM CTX-M	NDM CTX-M	…
Date of death	Sept 24, 2020	Sept 17, 2020	Sept 21, 2020	…
Date of discharge	…	…	…	Oct 6, 2020

*Klebsiella pneumoniae* MDR has become a threat to public health; by itself, it has virulence factors related to high mortality as well as low response to treatment.^3^ *Klebsiella pneumoniae* MDR was described for some time in Latin America and specifically in Peru in 2016, where an increased proportion of Enterobacteriaceae cases were carrying the NDM gene (67.5%) compared to *Klebsiella pneumoniae* carbapenemase (KPC)–producing bacteria (31.3%) and active-on-imipenem (IMP)–type NDM bacteria (1.2%).^4^ Sacsaquispe et al^5^ determined that the main mechanism of resistance to carbapenems is the expression of *bla*NDM carbapenemase.

The COVID-19 pandemic has been superimposed on another pandemic, that of MDR bacteria. In our case, our hospital capacity was overwhelmed by patient overcrowding, so we managed patients with moderate and severe disease in patient reception areas and outpatient clinics in addition to opening a provisional intensive care unit (ICU) for critical patients. Under normal conditions, ICUs are the epicenters for the development of MDR bacteria.6

Another mechanism facilitating MDR spread is the irrational use of antibiotics, which were given due to the initial suspicion of superimposed infections. International reports indicate that up to 70% of hospitalized COVID-19 patients receive antibiotics, and these are often broad-spectrum agents, despite results that indicate a low proportion of bacterial infections.^7^ For this reason, treatment guidelines do not recommend the use of antibiotics in patients with mild or moderate disease unless the suspicion of bacterial confection is important.^8^

Cross contamination via the hands of the staff would be the main means of transmission; unfortunately, no further audit was possible due to the work overload in all areas of our hospital. Furthermore, the overload of the health systems and staff burnout may have decreased adherence to infection prevention and control, which may have facilitated the spread of MDR germs.^9^

The limited and inappropriate use of PPE is another factor to consider.^2^ Due to the great demand for this equipment, we experienced deficiencies in its availability and have had only 1 set of PPE to be used with all patients and throughout the shift, which could have facilitated the spread of germs from staff to the patients.

The hospital overflow and the isolation of health personnel with risk factors had to be mitigated by hiring young personnel who were poorly trained in the management of infections associated with health care. Suggestions for controlling the spread of bacterial resistance in the context of the COVID-19 pandemic include increasing the competencies of physicians in the proper treatment of SARS-CoV-2, correct recognition of symptoms of superimposed infections, eliminating the unnecessary use of antibiotics, and assessing the need for the use of devices that are known to increase the probability of infection.^9^

In addition, when treating a patient presenting with severe pneumonia due to COVID-19, in a state of immunological depression and in need of mechanical ventilation, it is necessary to consider the possibility of coinfection, as occurred with 2 of our patients. Thus, it is important to search for other bacteria with equal or greater virulence because coinfections may exist in hospitalized patients with COVID-19.^10^

In conclusion, while we find ourselves in a pandemic state due to a virus that is not yet fully understood, COVID-19 patients have an even higher risk of acquiring MDR bacterial infections, leading to a mortality rate even higher than that conferred by COVID-19 alone.
